# Lung-Reporting and Data System Category (Lung-RADS) and Adherence to Low-Dose Computed Tomography Screening and Recommended Follow-Up: A Multicenter Cohort Study

**DOI:** 10.1016/j.jtocrr.2026.101077

**Published:** 2026-09-07

**Authors:** Pratibha Shrestha, Michael K. Gould, Gerard A. Silvestri, Xiangyang Lou, Christopher G. Slatore, Perry J. Pickhardt, Bruno Hochhegger, Himanshi Verma, Pianpian Cao, Jiang Bian, Dejana Braithwaite, Shama D. Karanth

**Affiliations:** a Division of Population Health Sciences, Department of Surgery, College of Medicine, University of Florida, Gainesville, Florida; b Department of Health Systems Science, Kaiser Permanente Bernard J. Tyson School of Medicine, Pasadena, California; c Department of Medicine, Medical University of South Carolina, Charleston, South Carolina; d Department of Biostatistics, University of Florida, Gainesville, Florida; e Center to Improve Veteran Involvement in Care, Portland VA Medical Center, Portland, Oregon; f Division of Pulmonary, Critical Care, and Allergy Medicine, Department of Medicine, Oregon Health & Science University, Portland, Oregon; g University of Wisconsin School of Medicine and Public Health, Madison, Wisconsin; h Department of Radiology, University of Florida College of Medicine, Gainesville, Florida; i University of Florida Health Cancer Institute, Gainesville, Florida; j University of Miami Leonard M. Miller School of Medicine, Miami, Florida; k Department of Public Health, College of Health and Human Sciences, Purdue University, West Lafayette, Indiana; l Department of Biostatistics and Health Data Science, Indiana University School of Medicine, Indianapolis, Indiana; m Regenstrief Institute, Indianapolis, Indiana; n Department of Epidemiology, University of Florida College of Public Health and Health Professions, Gainesville, Florida

**Keywords:** Adherence, Follow-up care, Frailty, Lung cancer screening, Low-dose computed tomography

## Abstract

**Background:**

Adherence to guideline-recommended low-dose computed tomography (CT) screening and follow-up remains suboptimal, and determinants may vary by Lung Imaging Reporting and Data System (Lung-RADS) category.

**Methods:**

This retrospective, multi-site cohort study used electronic medical records from adults aged 50 to 80 years. Eligible individuals had at least one primary care visit in the previous 12 months, no lung cancer diagnosis in the preceding 5 years, and underwent an index low-dose CT between 2016 and 2021. Participants were followed through 2022. Adherence to follow-up chest CT within 11 to 15 months (Lung-RADS 1–2) and category-specific recommended follow-up (Lung-RADS 3–4X) was assessed using mixed-effects logistic regression to estimate adjusted ORs (aORs) and 95% confidence intervals (CIs).

**Results:**

Among 22,034 individuals with Lung-RADS 1 to 2, 21.67% completed follow-up CT within 11 to 15 months. The odds of adherence were higher among individuals aged 65 or more years (versus <65) (aOR = 1.22; 95% CI: 1.14–1.31) and those with chronic obstructive pulmonary disease (COPD) (aOR = 1.18; 95% CI: 1.09–1.27) and lower among black (versus white) individuals (aOR = 0.87; 95% CI: 0.79–0.96) and frail (versus non-frail) individuals (aOR = 0.88; 95% CI: 0.80–0.98). Among 4518 individuals with Lung-RADS 3 to 4X, 28.51% adhered to category-specific follow-up; COPD was the only factor independently associated with adherence (aOR = 1.22; 95% CI: 1.06–1.40).

**Conclusion:**

Adherence to follow-up CT and recommended care remains low in real-world settings; older age and COPD predicted higher adherence, while lower adherence among black individuals identifies a key target for improving lung cancer screening retention.

## Introduction

Lung cancer is the leading cause of cancer-related mortality worldwide, accounting for approximately 124,730 deaths annually in the United States^1^^,^^2^ The US Preventive Services Task Force recommends annual low-dose computed tomography (LDCT) screening for adults aged 50 to 80 years with a 20 pack-year smoking history who currently smoke or have quit within the past 15 years.^3^ LDCT screening has been shown to reduce lung cancer-specific mortality by enabling detection at earlier, more treatable stages, provided that appropriate and timely follow-up care is completed.^4^^,^^5^

In clinical practice, the American College of Radiology Lung Computed Tomography Screening Reporting and Data System (Lung-RADS) guides follow-up care after baseline LDCT.^6^ Patients with negative results (Lung-RADS 1–2) are advised to continue annual screening, whereas those with positive results require follow-up imaging in 6 months (Lung-RADS 3) or a more intensive evaluation (Lung-RADS 4).^4^^,^^6^ Despite these guidelines and the potential benefits of lung cancer screening (LCS),^7^ real-world adherence to LCS remains suboptimal.^4^^,^^8^, ^9^, ^10^ Screening uptake remains low, with fewer than 20% of eligible individuals undergoing LCS in U.S. populations.^11^ Among those who initiate screening, adherence to annual repeat screening remains suboptimal, with estimates generally ranging from approximately 22% to 57% across studies.^12^^,^^13^ In contrast, follow-up after a positive screening result varies widely, with higher adherence reported in integrated systems, such as the Veterans Health Administration (∼63%).^14^ These rates remain substantially lower than the approximately 95% adherence achieved in clinical trial settings, such as the National Lung Screening Trial.^5^ Importantly, microsimulation modeling suggests that the effectiveness of LCS is highly sensitive to adherence; estimated lung cancer deaths decrease from 501 to 175 per 100,000 persons as annual adherence declines from 100% to 29%.^15^

The observed low adherence to recommended LCS follow-up is concerning, as consistent participation is essential for early detection and a reduction in mortality.^4^ Multiple factors may contribute to lower adherence, including patient-level demographics, contextual factors, and clinical characteristics. Prior studies report lower adherence among younger individuals,^13^ Black patients compared with white patients, and populations facing socioeconomic disadvantage.^16^ Women are more likely to receive follow-up care than men,^9^ and additional factors such as body mass index (BMI), education, and history of cancer may also influence adherence.^13^ Frailty better reflects functional reserve than comorbidity alone,^17^ yet its role in LCS adherence and follow-up remains underexplored.^18^ Low adherence may, among other factors, reflect the inclusion of patients with limited life expectancy or a high comorbidity burden-populations typically excluded from clinical trials.^19^ Although comorbidities are known to reduce LCS uptake,^20^ frailty represents a distinct age-related vulnerability characterized by reduced physiologic reserve and an increased risk of adverse outcomes.^21^ Prior studies have reported that frailty is common among older adults with newly diagnosed lung cancer, particularly among those aged 70 or more years.^22^^,^^23^

Although prior studies have evaluated adherence to LCS and follow-up, important gaps remain, particularly in real-world, multi-site populations and across Lung-RADS categories. The multi-center personalized lung screening cohort addresses these gaps by providing a comprehensive, pragmatic assessment of adherence to both annual screening—defined as completion of any CT within a flexible interval of 11 to 15 months—and guideline-recommended follow-up, allowing a ±1 month, or flexible, window around Lung-RADS-specific timeframes across diverse clinical settings. To better reflect real-world variability in care delivery, we conducted sensitivity analyses using broader follow-up windows, including extended annual screening intervals (11–24 months) and expanded Lung-RADS-specific follow-up periods. By integrating functional status with patient-level and contextual factors, this study aims to inform more personalized and effective implementation of LCS programs.

## Materials and Methods

### Study Population

The study population was drawn from the previously described personalized lung screening cohort, a multicenter study designed to evaluate the real-world implementation and outcomes of LDCT screening to inform personalized screening strategies.^24^ Individuals were identified from electronic health records (EHR) and institutional or state cancer registries across the University of Florida (UF), Kaiser Permanente Southern California, and the Medical University of South Carolina. We included adults who underwent baseline or repeat annual LDCT, identified via current procedural terminology (CPT) and healthcare common procedure coding system (HCPCS) codes G0297, S8032, and 71271, between 2016 and 2021, with follow-up through 2022.^24^ Eligible individuals had 1 or more primary care visit within 12 months before their baseline screening date and no history of lung cancer in the preceding 5 years, which was determined using linked cancer registry data available through the UF Health Integrated Data Repository. The UF Integrated Data Repository integrates electronic health record and tumor registry information. A single institutional review board at Kaiser Permanente Southern California provided oversight for the study, with UF and Medical University of South Carolina ceding review authority. The institutional review board reviewed and approved the study and determined that informed consent was not required because this retrospective observational analysis used existing data.

Of the N = 31,795 patients initially identified, we excluded patients younger than 50 or older than 80 years (n = 1053), consistent with the US Preventive Services Task Force LCS recommendation.^3^ We further excluded patients with unknown or incomplete index Lung-RADS scores (n = 1255) and those who had never smoked or had an unknown smoking history (n = 2475). The remaining cohort (n = 27,012) was categorized into negative Lung-RADS^1^^,^^2^ and positive Lung-RADS 3 through 4X groups based on their index LDCT results. Among the negative Lung-RADS results, we excluded those diagnosed with lung cancer (n = 67) within 15 months of the index LDCT and those who died within 15 months (n = 353). Among the positive Lung-RADS results, we excluded those who were still within the recommended follow-up interval based on Lung-RADS category: within 210 days for Lung-RADS 3, within 120 days for Lung-RADS 4A, and within 45 days for Lung-RADS 4B and 4X (n = 40) (Fig. 1).

**Figure 1:**
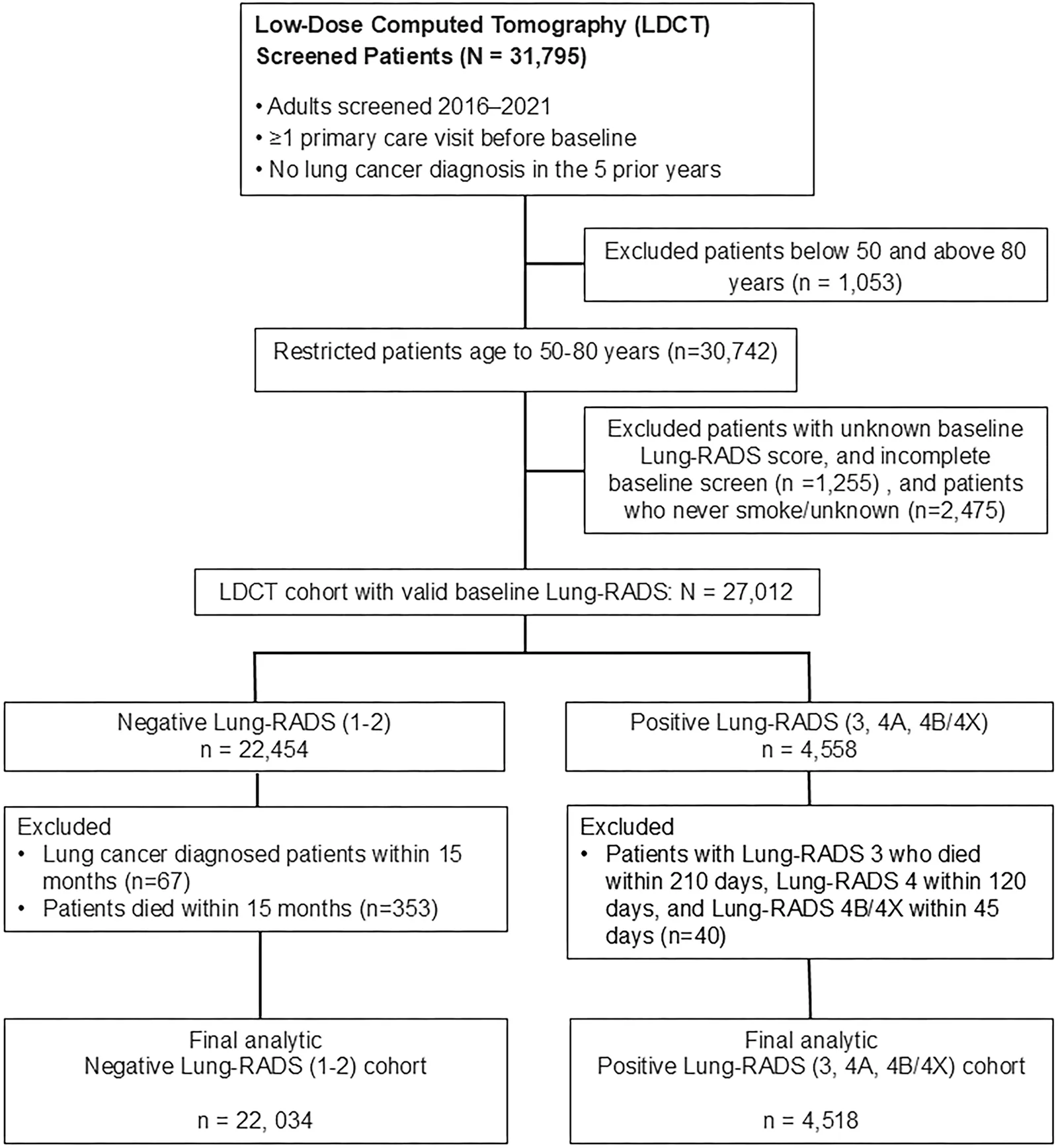
Flow diagram for the inclusion and exclusion criteria for assessment of adherence and follow-up care.

### Exposures and Outcome of Interest

Exposures of interest were selected a priori and included baseline demographic, socioeconomic, and clinical characteristics. Age was categorized as less than 65 years, 65 years or more; sex as male or female; race as white, black, Asian, other, or unknown; and ethnicity as non-Hispanic, Hispanic, or unknown. Marital status was classified as never married, married/living as married, previously married (widowed/separated/divorced), or unknown.

Socioeconomic context was measured using census-tract area-level educational attainment, defined as the proportion of adults in the patient’s neighborhood with a college degree, and categorized as 0% to 19.9%, 20% to 39.9%, 40% or higher, and unknown/missing. BMI (kg/m^2^) was categorized as underweight (<18.5), normal weight (18.5 to <25), overweight (25 to <30), obese (≥30), and unknown. Smoking status was categorized as current or former.

Clinical exposures included a history of prior cancer (excluding lung cancer and non-melanoma skin cancer) within 2 years before the index date and chronic obstructive pulmonary disease (COPD) identified using International Classification of Diseases-9 and International Classification of Diseases-10 codes, categorized as yes/no. Frailty was assessed using a validated claims-based frailty index derived from inpatient and outpatient claims during the 1-year period preceding the baseline LDCT, constructed using a previously described SAS macro.^25^^,^^26^ The index incorporates diagnoses and utilization markers reflecting functional vulnerability (e.g., impaired mobility, cognitive impairment, depression, falls, and infections) along with comorbidity and utilization measures (e.g., inpatient admissions and Charlson Comorbidity Index) and yields a score ranging from 0 to 1, with higher values indicating greater frailty.^25^^,^^26^ Frailty was defined as a score of 0.2 or higher, consistent with prior literature.^25^ The screening site was included as a covariate to account for differences in care processes and patient populations.

Our primary outcomes were adherence to (1) CT-based follow-up after an index negative Lung-RADS 1 to 2 result and (2) recommended follow-up care after an index positive Lung-RADS 3–4X score (Table 1).^8^^,^^10^ The index Lung-RADS category was obtained from standardized screening data collected across participating sites and reflected the category assigned at the time of the index LDCT examination (Lung-RADS 0, 1, 2, 3, 4A, 4B, or 4X). Examinations were not retrospectively reread or reclassified by the study team; analyses were based on the Lung-RADS category recorded in the source data. Lung-RADS management recommendations specify annual LDCT screening for Lung-RADS 1to 2, 6-month follow-up imaging for Lung-RADS 3, 3-month follow-up imaging with consideration of positron emission tomography (PET)/CT for Lung-RADS 4A, and prompt diagnostic evaluation with chest CT, PET/CT, and/or tissue sampling for Lung-RADS 4B/4X findings.^27^ For negative Lung-RADS 1 to 2 findings, adherence was defined as completion of CT-based imaging, including LDCT, diagnostic chest CT, or PET/CT, within 11 to 15 months after the index LDCT. This window was selected to capture follow-up around the recommended 12-month annual screening interval while allowing a clinically reasonable grace period. Patients who did not complete any qualifying CT-based imaging during this timeframe were classified as nonadherent.

**Table 1:** Category-Specific Definitions of Adherence for Lung-RADS Negative^1^^,^^2^ and Lung-RADS Positive (3, 4A, 4B/4X) Results

Lung-RADS Category	Follow-up Window	Follow-up Modality	Adherent Definition
Lung-RADS 1–2 (n = 22,034)	11–15 mo	LDCT or diagnostic CT/PET-CT	Completion of any chest CT or PET/CT within 11–15 mo after index LDCT
Lung-RADS 3 (n = 2040)	150–210 d	Chest CT	Completion of chest CT within 150–210 d after index LDCT
Lung-RADS 4A (n = 1542)	60–120 d or ≤ 45 d (biopsy)	Chest CT or PET/CT or biopsy (bronchoscopy, percutaneous biopsy, surgical resection)	Completion of chest CT or PET/CT within 60–120 d, or biopsy within 45 d after index LDCT
Lung-RADS 4B/4X (n = 936)	≤ 45 d	Chest CT, or PET/CT or Biopsy (bronchoscopy, percutaneous biopsy, surgical resection)	Completion of chest CT, PET/CT, or biopsy within 45 d after index LDCT

Adherence windows were operationalized using clinically informed time ranges to reflect real-world variation in follow-up timings. For Lung-RADS 1–2, “any CT” includes low-dose CT (LDCT) as well as clinically indicated diagnostic CT or PET/CT. Time windows approximate Lung-RADS recommendations (12 months, 6 months, 3 months, and immediate follow-up) with allowance for early and delayed care. CT, computed tomography; LDCT, low-dose CT; Lung-RADS, Lung Imaging Reporting and Data System; PET, positron emission tomography.

For positive Lung-RADS 3 to 4X findings, adherence was defined using category-specific follow-up windows based on Lung-RADS management recommendations and prior LCS adherence literature.^8^^,^^27^ For Lung-RADS 3, adherence was defined as completion of chest CT within 150 to 210 days after the index LDCT, corresponding to a ±1-month window around the recommended 6-month follow-up interval. For Lung-RADS 4A, adherence was defined as completion of chest CT or PET/CT within 60 to 120 days after the index LDCT, corresponding to a ±1-month window around the recommended 3-month follow-up interval, or completion of biopsy within 45 days after the index LDCT. For Lung-RADS 4B/4X, adherence was defined as completion of chest CT, PET/CT, or biopsy, including bronchoscopy, percutaneous biopsy, or surgical resection, within 45 days after the index LDCT. Despite these category-specific definitions, adherence across Lung-RADS 3, 4A, and 4B/4X was combined into a single binary outcome for the primary regression analysis evaluating factors associated with recommended follow-up after any positive screening result.

### Statistical Analysis

Descriptive frequency tables were generated for demographic, clinical, and behavioral characteristics stratified by Lung-RADS category from the index LDCT (negative and positive). Mixed-effects logistic regression models with a random intercept for site were used to estimate adjusted ORs (aORs) and 95% confidence intervals (CIs). Models were fitted using adaptive Gauss-Hermite quadrature (QPOINTS = 7), consistent with recommended practices in the literature.^28^, ^29^, ^30^ We evaluated demographic, socioeconomic, and clinical factors associated with (1) adherence to any CT screening among patients with Lung-RADS 1 to 2 and (2) adherence to guideline-recommended follow-up among patients with Lung-RADS 3 to 4X. All analyses, including sensitivity analyses, were conducted using SAS (version 9.4; SAS Institute Inc., Cary, NC). Figures were generated using Microsoft PowerPoint and Python with NumPy and Matplotlib and were finalized in Adobe Photoshop.

For sensitivity analyses, we broadened the primary adherence definition windows to assess the robustness of the findings under alternative follow-up definitions. The follow-up modalities remained the same as in the primary analysis, but the allowable time windows were extended. Among patients with negative index findings, Lung-RADS 1 to 2 adherence was redefined as completion of any subsequent CT-based imaging within 11 to 24 months after the index LDCT. Among those with positive index findings, extended windows were defined by Lung-RADS category: Lung-RADS 3 adherence was defined as completion of follow-up chest CT within 120 to 270 days after the index LDCT (±2 mo around the recommended 6-mo interval); Lung-RADS 4A adherence was defined as completion of chest CT or PET/CT within 30 to 150 days or biopsy within 150 days after the index LDCT, reflecting an extended window around the recommended 3-month follow-up interval; and Lung-RADS 4B/4X adherence was defined as completion of chest CT, PET/CT, or biopsy within 150 days after the index LDCT. As an additional sensitivity analysis, we fit separate models within the Lung-RADS 3 to 4X cohort, stratified by index Lung-RADS category, including Lung-RADS 3, Lung-RADS 4A, and Lung-RADS 4B/4X, using the primary follow-up definitions (Supplementary Table 1). We also evaluated broader adherence windows to assess the robustness of the findings under alternative follow-up definitions; corresponding analytic denominators are reported in Supplementary Table 2.

## Results

Of 31,795 patients, 6279 were excluded, leaving 22,034 patients with Lung-RADS negative findings^1^^,^^2^ and 4518 patients with Lung-RADS positive findings (3 to 4X) at the index LDCT (Fig. 1). In the Lung-RADS 1 to 2 group (Table 2), the mean age was 64.26 years. Most patients were white (67.37%), followed by black (15.50%) and Asian (7.62%). More than half were current smokers (52.88%), whereas 47.12% were former smokers. Among patients with available smoking histories, the median pack-years was 30.00 (IQR = 15.00–40.00), with detailed pack-year histories available for approximately 70.00% of participants. The prevalence of COPD was 28.80%, and 15.54% were frail. In the Lung-RADS 3–4X group, the mean age was 66.15 years. Most patients were white (71.45%), followed by black (12.42%) and Asian (7.26%). Current smokers represented 50.84% of the group, whereas 49.16% were former smokers. Among patients with available smoking histories, the median pack-years was 30.00 (IQR = 17.50–44.00). The prevalence of COPD was 37.54%; 48.01% were married or living as married, 2.61% had a history of prior cancer, and 22.53% were frail (Table 2).

**Table 2:** Distribution of Patients Characteristics by Lung-RADS Negative^1^^,^^2^ and Lung-RADS Positive (3, 4A, 4B/4X) categories

Variables	Lung RADS^1^^,^^2^ (N = 22,034)	Lung RADS (3, 4A, 4B/4X) (N = 4518)
Age, mean (SD), y	64.26 (6.51)	66.15 (6.63)
Age group, y, n (%)		
<65	11,641 (52.83)	1826 (40.42)
≥65	10,393 (47.17)	2692 (59.58)
Sex, n (%)		
Male	12,772 (57.96)	2754 (60.96)
Female	9262 (42.04)	1764 (39.04)
Race, n (%)		
White	14,844 (67.37)	3228 (71.45)
Black	3415 (15.50)	561 (12.42)
Asian	1680 (7.62)	328 (7.26)
Others	230 (1.04)	48 (1.06)
Unknown	1865 (8.46)	353 (7.81)
Ethnicity, n (%)		
Non-Hispanic	16,774 (76.13)	3467 (76.74)
Hispanic	3042 (13.81)	553 (12.24)
Unknown/Missing	2218 (10.07)	498 (11.02)
Marital status, n (%)		
Never married	4024 (18.26)	774 (17.13)
Married/Living as married	10,547 (47.87)	2169 (48.01)
Previously married (Widowed/Separated/Divorced)	4050 (18.38)	771 (17.07)
Unknown/Missing	3413 (15.49)	804 (17.80)
Area education status, n (%)		
0%–19.9% have college degree	10,595 (48.08)	2357 (52.17)
20%–39.9% have college degree	7989 (36.26)	1663 (36.81)
>40% have college degree	1556 (7.06)	273 (6.04)
Unknown/Missing	1894 (8.60)	225 (4.98)
Body mass index, n (%)		
Underweight (<18.5)	468 (2.12)	156 (3.45)
Normal weight (18.5 to < 25)	5846 (26.53)	1405 (31.10)
Overweight (25 to <30)	7550 (34.27)	1465 (32.43)
Obesity (≥30)	7357 (33.39)	1339 (29.64)
Unknown	813 (3.69)	153 (3.39)
Smoking status, n (%)		
Current smoker	11,651 (52.88)	2297 (50.84)
Former smoker	10,383 (47.12)	2221 (49.16)
Smoking pack-years		
Median (IQR)	30 (15–40)	30 (17.5–44)
Missing n (%)	6656 (31.29)	1342 (31.64)
History of prior cancer, n (%)		
No	21,523 (97.68)	4400 (97.39)
Yes	511 (2.32)	118 (2.61)
COPD, n (%)		
No	15,688 (71.20)	2822 (62.46)
Yes	6346 (28.80)	1696 (37.54)
Frailty, n (%)		
Non-Frail (<0.2)	18,937 (86.04)	3500 (77.47)
Frail (≥0.2)	2338 (10.61)	1018 (22.53)

COPD, chronic obstructive pulmonary disease; IQR, interquartile range**;** Lung-RADS, Lung Imaging Reporting and Data System; SD, standard deviation.

Among 22,034 patients with Lung-RADS 1 to 2 findings, overall adherence to CT screening within 11 to 15 months was 21.67% (95% CI: 21.12%–22.22%). In adjusted mixed-effects models, the odds of adherence were higher among individuals aged 65 years or more than among those less than 65 years (aOR = 1.22; 95% CI: 1.14–1.31), among those with overweight BMI than among those with normal weight (aOR = 1.10; 95% CI: 1.01–1.20), and among those with COPD than among those with no COPD (aOR = 1.18; 95% CI: 1.09–1.27). The odds of adherence were lower among black patients than among white patients (aOR = 0.87; 95% CI: 0.79–0.96) and among frail individuals than among non-frail individuals (aOR = 0.88; 95% CI: 0.80–0.98). Smoking status, sex, marital status, area-level education, and prior cancer history were not significantly associated with adherence (Fig. 2).

**Figure 2:**
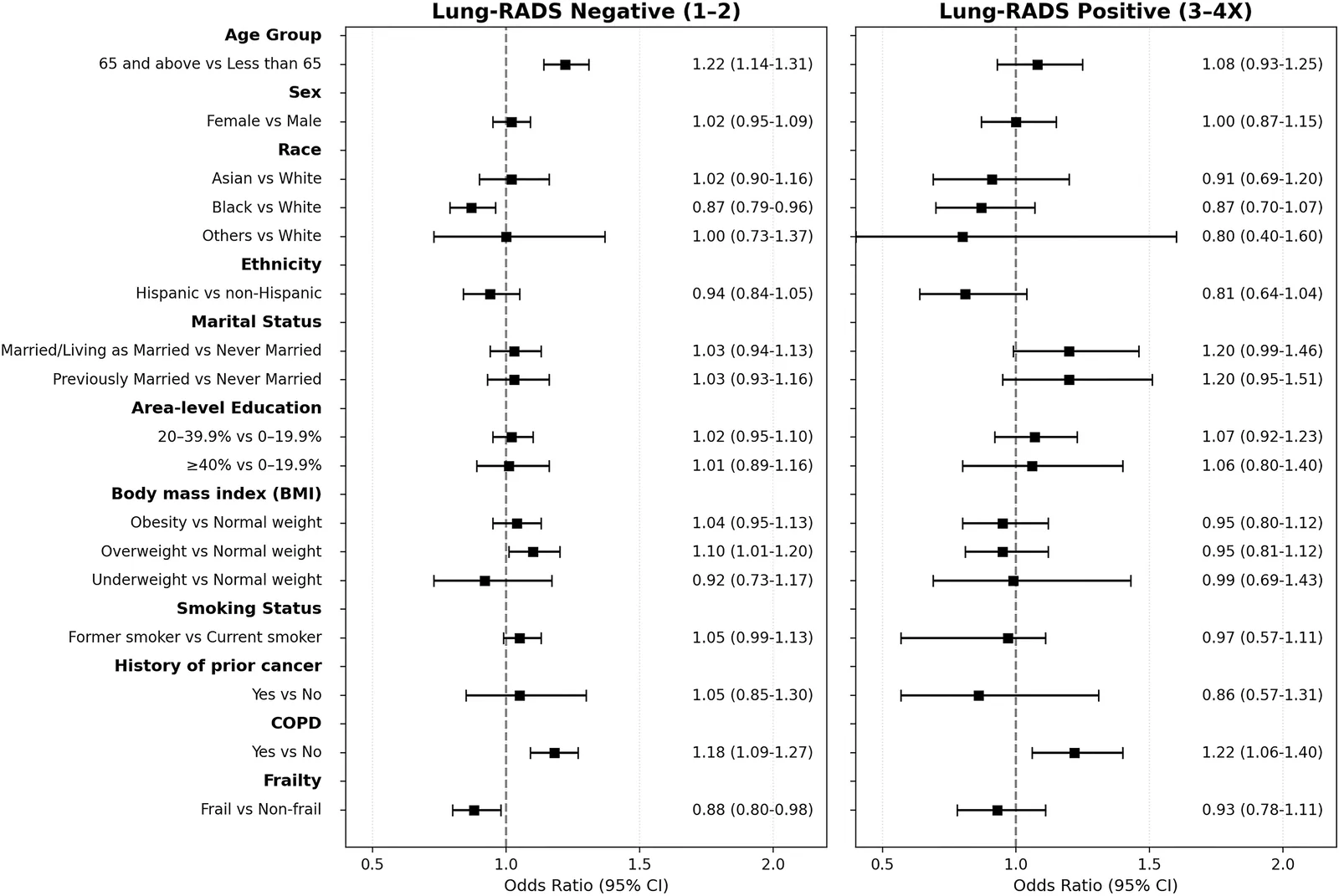
Factors Associated with Adherence to CT Follow-up in Lung-RADS Negative^1^^,^^2^ and Positive (3–4X). Models were adjusted for age group, sex, race, ethnicity, marital status, neighborhood education, BMI, smoking status, history of prior cancer, COPD, and frailty, and included a random intercept for site. Unknown or missing categories were included in the models but are not displayed. Adherence was defined as completion of guideline-recommended follow-up after the index LDCT within the specified window for each Lung-RADS category: for Lung-RADS categories 1 to 2, completion of any chest CT (LDCT, diagnostic CT, or PET/CT) within 11 to 15 months; for Lung-RADS category 3, completion of chest CT within 150–210 days; for Lung-RADS category 4A, completion of chest CT or PET/CT within 60 to 120 days or biopsy within 45 or less days; and for Lung-RADS categories 4B/4X, completion of chest CT, PET/CT, or biopsy within 45 or less days. For the regression analysis, adherence among Lung-RADS categories 3–4X was modeled as a single combined binary outcome. BMI, body-mass index; COPD, chronic obstructive pulmonary disease; CT, computed tomography; LDCT, low-dose CT; Lung-RADS, Lung Imaging Reporting and Data System; PET, positron emission tomography.

Among 4518 patients with Lung-RADS 3 to 4X findings, 28.51% (95% CI: 27.19%–29.85%) adhered to follow-up care within category-specific time windows (Lung-RADS 3, 150–210 d; Lung-RADS 4A, 60–120 d; Lung-RADS 4B/4X, ≤45 d; Table 1). Adherence was 24.61% (95% CI: 22.75%–26.54%) for Lung-RADS 3, 28.73% (95% CI: 26.48%–31.06%) for Lung-RADS 4A, and 36.65% (95% CI: 33.55%–39.82%) for Lung-RADS 4B/4X. Among 298 individuals diagnosed with lung cancer within 1 year of a positive Lung-RADS result, 69.13% completed guideline-concordant follow-up testing. In adjusted models, COPD was associated with higher odds of completing timely follow-up (aOR = 1.22; 95% CI: 1.06–1.40), whereas race, ethnicity, marital status, area-level education, BMI, smoking status, prior cancer history, and frailty were not significantly associated with adherence (Fig. 2).

In stratified analysis by positive Lung-RADS category, the association between age and adherence differed across severity levels (Supplementary Table 1). Among patients with Lung-RADS 3 findings, individuals aged 65 or more years had lower odds of adherence compared with those aged less than 65 years (aOR = 0.78; 95% CI: 0.62–0.99). In contrast, among patients with Lung-RADS 4A findings, older age was associated with higher odds of adherence (aOR = 1.32; 95% CI: 1.01–1.71). A similar, although non-significant, positive association was observed among Lung-RADS 4B/4X patients (aOR = 1.22; 95% CI: 0.89–1.67) (Supplementary Table 1).

### Sensitivity Analyses

Broadening adherence windows yielded results generally consistent with the primary analysis. Among patients with Lung-RADS 1 to 2 findings, extending the screening interval to 11 to 24 months increased adherence to 33.40% (95% CI: 32.77%–34.02%). In adjusted models, age 65 or more years, compared with less than 65 years (aOR = 1.24; 95% CI: 1.16–1.32), and COPD remained associated with higher odds of adherence, whereas black race, compared with white race, was associated with lower odds of adherence. Frail individuals were associated with lower odds of adherence than non-frail individuals (aOR = 0.84; 95% CI: 0.77–0.92) (Supplementary Table 2).

Among patients with Lung-RADS 3 to 4X findings, overall follow-up adherence using the extended window was 45.41% (95% CI: 43.94%–46.88%). Adherence varied by Lung-RADS category, increasing from 39.41% (95% CI: 37.28%–41.57%) among Lung-RADS 3 patients to 45.08% (95% CI: 42.56%–47.61%) among Lung-RADS 4A patients and 59.26% (95% CI: 56.00%–62.46%) among Lung-RADS 4B/4X patients. In adjusted models, older age (≥ 65 years) was associated with higher odds of adherence (aOR = 1.20; 95% CI: 1.05–1.38) compared with younger individuals (<65 years). Obesity, compared with normal weight (aOR = 0.79; 95% CI: 0.67–0.92), and overweight, compared with normal weight (aOR = 0.84; 95% CI: 0.72–0.98), were associated with lower odds of adherence. Other variables, including smoking status, COPD, frailty, and other sociodemographic factors, were not significantly associated with adherence (Supplementary Table 2). Crude adherence rates varied across participating study sites and are presented in Supplementary Table 3.

## Discussion

This study identified important differences in adherence to LDCT follow-up across Lung-RADS categories, with overall adherence remaining suboptimal. Among patients with Lung-RADS 1 to 2 findings, only 21.67% adhered to repeat screening within 11 to 15 months, increasing modestly to 33.40% when the interval was extended to 11 to 24 months. These findings are consistent with the low uptake (22.30%) reported in the American College of Radiology LCS Registry.^12^ In this Lung-RADS 1 to 2 group, older age and the presence of COPD were associated with greater adherence to follow-up CT screening, suggesting that a higher perceived clinical risk may motivate patients to return for screening.^31^ In contrast, frailty was associated with lower LCS adherence, which may reflect both physical and functional limitations that make follow-up imaging more difficult and clinical decision-making about the expected benefit of continued LCS among frail individuals.^19^^,^^23^

Follow-up after a positive Lung-RADS result was also low, with 28.51% adhering to our primary defined timeframe, increasing to 45.41% using the extended window. Under the extended definition, adherence varied by Lung-RADS category, increasing from 39.41% among Lung-RADS 3 to 45.08% among Lung-RADS 4A and 59.26% among Lung-RADS 4B/4X. In our primary analysis, adherence rates were 24.61% for Lung-RADS 3, 28.73% for Lung-RADS 4A, and 36.65% for Lung-RADS 4B/4X. These rates were lower than those reported by Rivera et al.,^8^ who defined adherence using American College of Radiology-recommended follow-up timing for Lung-RADS 3 and 4A, and a 4-week follow-up window for Lung-RADS 4B/4X because American College of Radiology Lung-RADS does not specify an exact timeframe for these higher-risk findings. In their cohort, adherence was 30% for Lung-RADS 3, 49.50% for Lung-RADS 4A, and 68% for Lung-RADS 4B/4X, with higher adherence when extended windows were applied. Rivera et al.^8^ evaluated follow-up after positive screening results in a smaller cohort from academic and community screening programs with structured tracking processes, whereas our study included a larger, multi-site, real-world population with more heterogeneous care delivery and patient complexity. Our primary adherence definitions also used clinically informed bounded windows approximating Lung-RADS recommendations, such as 150 to 210 days for Lung-RADS 3, corresponding to the recommended 6-month follow-up interval with an approximately ±1-month grace period. Triplette et al.^32^ reported 47% adherence in an academic multisite LCS program, where follow-up may have been supported by more structured screening workflows and stronger program-level coordination than is typical in routine real-world care.^32^ Unlike our study, which focused on completion of the initial category-specific recommended follow-up after an index positive LDCT, Kim et al.^4^ evaluated annual repeat screening adherence across five health systems using broader longitudinal windows and shifted timing for patients with positive index results. Kim et al.^4^ reported annual adherence of 61.20% at the first follow-up interval and 50.50% at the second follow-up interval, which is therefore more comparable to repeat annual screening than to immediate diagnostic follow-up after positive Lung-RADS findings.^4^ These discrepancies likely reflect differences in sample size, study setting, patient population, care coordination, adherence definitions, program infrastructure, and patient complexity in routine practice.

Among patients with Lung-RADS 3 to 4X findings, COPD was the only factor significantly associated with adherence in the combined positive Lung-RADS model, suggesting that patients with underlying pulmonary disease may receive greater clinical attention, have more frequent health care contact, or have a stronger linkage to pulmonary care. In addition, individuals with COPD may perceive themselves to be at higher risk for lung cancer, which has been associated with greater engagement in screening-related behaviors and follow-up care.^33^ In supplementary analyses stratified by positive Lung-RADS category, the association between age and adherence varied by Lung-RADS severity. Older age was associated with lower adherence among Lung-RADS 3 patients but higher adherence among Lung-RADS 4A patients, whereas a similar but nonsignificant positive pattern was observed among Lung-RADS 4B/4X patients. This pattern may reflect differences in perceived urgency and clinical prioritization: Lung-RADS 3 findings require 6-month follow-up and may be perceived as having lower urgency, whereas Lung-RADS 4A findings require shorter-interval follow-up and may prompt stronger clinician communication and greater perceived cancer risk.^8^^,^^27^

Notably, among individuals diagnosed with lung cancer within 1 year of a positive Lung-RADS result, adherence to follow-up testing was substantially higher (69.13%), suggesting that engagement with care increases once disease is clinically confirmed or strongly suspected. Together, these findings highlight a persistent gap between guideline-recommended follow-up and real-world LCS practice. Adherence varied by Lung-RADS category and patient characteristics, suggesting that both clinical risk perception and care delivery processes influence follow-up completion. Racial disparities were also evident, with lower adherence among black compared with white patients, consistent with well-documented racial disparities in LCS.^10^^,^^34^

Clinically, COPD was associated with higher adherence, likely reflecting more frequent healthcare contact and greater involvement of pulmonary specialists,^35^ heightened perceived risk of lung cancer,^33^ and its inclusion in risk-based screening models (e.g., PLCOm2012), which may increase clinical prioritization for follow-up.^36^ Overweight status was also associated with better adherence, potentially reflecting greater healthcare engagement.^37^ In contrast, studies using claims and EHR data have reported that comorbid conditions, insurance status, and health-related social needs, including housing instability and transportation barriers, contribute to missed imaging appointments and delayed follow-up.^8^^,^^32^^,^^38^ Disparities are often more pronounced in decentralized screening programs, where adherence tends to be lower compared with centralized systems that incorporate tracking and coordination mechanisms.^10^ These findings underscore the importance of system-level factors, including access, care coordination, and follow-up infrastructure, in shaping adherence.^39^ Collectively, these results highlight the need for targeted interventions to reduce health disparities such as centralized tracking systems, patient navigation, reminders, and risk-stratified outreach to improve adherence in real-world LCS programs.^40^^,^^41^

Sensitivity analyses confirmed the robustness of these findings: although extended adherence windows increased overall rates, the pattern of associations remained unchanged. Sociodemographic and clinical factors, including age, race, BMI, COPD, and frailty, consistently influenced adherence.

Strengths of this study include its large, real-world, multi-site cohort and the use of mixed-effects models to account for site-level clustering. However, several limitations warrant consideration. First, reliance on EHR and administrative data may have led to misclassification of adherence, although this was mitigated by incorporating follow-up dates and diagnostic procedures and excluding patients who died during follow-up. Missing data, particularly for smoking pack-years, reflects the limitations of real-world documentation and may result in residual confounding. Adherence may have been underestimated when follow-up care occurred outside participating health systems, particularly among patients lost to follow-up. Differences in healthcare system structure and completeness of follow-up ascertainment across sites may also have contributed to between-site variation in adherence estimates. In addition, patient-reported and system-level factors that may influence adherence, such as transportation barriers and scheduling access, were not available. Adherence was defined based on completion of guideline-recommended imaging and biopsy procedures; care processes outside Lung-RADS follow-up recommendations, including pulmonology referrals, specialty consultations, and multidisciplinary management, were not considered. In addition, the claims-based frailty index may have been affected by incomplete capture of healthcare utilization outside participating health systems, and the exclusion of patients who died during follow-up may have influenced associations involving frailty.

Overall, our study findings have important clinical and public health implications. Incorporating frailty and comorbidity assessments into LCS eligibility may help avoid exposing vulnerable patients to low-value screening or follow-up.^20^^,^^42^, ^43^, ^44^ Targeted strategies, including patient navigation, reminders, and centralized tracking, are needed to improve adherence, particularly following positive screening results.^7^^,^^45^ Addressing structural and behavioral barriers, including health literacy, access, and risk perception, is critical to reducing adherence gaps. Future research is warranted to intervene among vulnerable populations, while also promoting screening and follow-up among younger eligible individuals (50–64 y), as adherence efforts should not be limited to older adults. Implementation science approaches are also needed to improve and optimize screening workflows in real-world practice. Emerging artificial intelligence-based methods for opportunistic frailty assessment from LDCT imaging may further support risk stratification when clinical measures are unavailable.^46^^,^^47^

In summary, adherence to LCS is critical to reducing mortality. A modeling study suggests that even moderate reductions in adherence substantially diminish the mortality benefit of screening.^15^ Despite using liberal adherence definitions, the rates in this study remained below the levels needed to achieve optimal population benefit. Improving adherence should therefore be a central priority for LCS programs to realize their full public health impact.

## CRediT Authorship Contribution Statement

**Pratibha Shrestha:** Conceptualization, Data curation, Formal analysis, Investigation, Methodology, Validation, Visualization, Writing - original draft, Writing - review & editing.

**Michael K. Gould:** Conceptualization, Funding acquisition, Investigation, Resources, Supervision, Writing - review & editing.

**Gerard A. Silvestri:** Funding acquisition, Investigation, Resources, Writing - review & editing.

**Xiangyang Lou:** Methodology, Supervision, Writing - review & editing.

**Christopher G. Slatore:** Resources, Writing - review & editing.

**Perry J. Pickhardt:** Writing - review & editing.

**Bruno Hochhegger:** Writing - review & editing.

**Himanshi Verma:** Writing - review & editing.

**Pianpian Cao:** Writing - review & editing.

**Jiang Bian:** Writing – review & editing.

**Dejana Braithwaite:** Conceptualization, Funding acquisition, Investigation, Methodology, Project administration, Resources, Supervision, Writing - review & editing.

**Shama D. Karanth:** Methodology, Project administration, Supervision, Writing - review & editing.

Dejana Braithwaite, Pratibha Shrestha, and Shama D Karanth had full access to all the study data and take responsibility for the integrity of the data and the accuracy of the data analysis.

## Disclosure

Dr. Gould reported personal fees from UpToDate for co-authorship of topics on lung cancer diagnosis and staging outside the submitted work. Dr. Silvestri reported grants from Nucleix, Delfi, and Freenome for screening biomarker work and personal fees from Biodesix outside the submitted work. The remaining authors declare no conflict of interest.
