# Supplementary material for: Lung-Reporting and Data System Category (Lung-RADS) and Adherence to Low-Dose Computed Tomography Screening and Recommended Follow-Up: A Multicenter Cohort Study
Source: JTO Clin Res Rep. 2026 Sep 7;7(11):101077. doi: 10.1016/j.jtocrr.2026.101077 (PMC13691248; doi:10.1016/j.jtocrr.2026.101077)
Supplement: Supplementary file 1 [file mmc1.docx]

**Supplementary Table S1:** Factors associated with adherence to guideline-recommended follow-up, stratified by Lung-RADS categories (3, 4A, and 4B/4X)

| **Variables** | **Lung-RADS 3** | **Lung-RADS 4A** | **Lung-RADS 4B/4X** |
| --- | --- | --- | --- |
|  | **(n=2,040)** | **(n=1,542)** | **(n=936)** |
|  | **aOR (95% CI)** | **aOR (95% CI)** | **aOR (95% CI)** |
| **Age Group** |  |  |  |
| 65 and above vs Less than 65 | 0.78 (0.62–0.99) | 1.32 (1.01–1.71) | 1.22 (0.89–1.67) |
| **Sex** |  |  |  |
| Female vs Male | 0.90 (0.72–1.13) | 0.94 (0.74–1.20) | 1.42 (1.06–1.92) |
| **Race** |  |  |  |
| Asian vs White | 1.11 (0.73–1.71) | 0.73 (0.45–1.18) | 0.85 (0.49–1.51) |
| Black vs White | 1.07 (0.77–1.48) | 0.85 (0.58–1.23) | 0.68 (0.44–1.06) |
| Others vs White | 0.81 (0.29–2.21) | 1.06 (0.27–4.09) | 0.66 (0.17–2.54) |
| **Ethnicity** |  |  |  |
| Hispanic vs non-Hispanic | 0.69 (0.47–1.02) | 0.83 (0.55–1.26) | 0.95 (0.56–1.61) |
| **Marital Status** |  |  |  |
| Married/Living as Married vs Never Married | 1.27 (0.94–1.73) | 1.18 (0.85–1.65) | 1.12 (0.75–1.68) |
| Previously Married vs Never Married | 1.46 (1.02–2.09) | 1.32 (0.89–1.97) | 0.75 (0.46–1.23) |
| **Area-level Education** |  |  |  |
| 20–39.9% vs 0–19.9% | 1.00 (0.79–1.26) | 0.96 (0.75–1.23) | 1.34 (0.99–1.82) |
| ≥40% vs 0–19.9% | 1.25 (0.82–1.91) | 0.95 (0.58–1.57) | 1.03 (0.54–1.96) |
| **Body mass index (BMI)** |  |  |  |
| Obesity vs Normal weight | 1.02 (0.78–1.34) | 1.07 (0.79–1.45) | 0.88 (0.61–1.28) |
| Overweight vs Normal weight | 0.99 (0.76–1.30) | 1.05 (0.75–1.47) | 0.92 (0.65–1.30) |
| Underweight vs Normal weight | 0.92 (0.47–1.82) | 0.76 (0.39–1.47) | 1.32 (0.69–2.51) |
| **Smoking Status** |  |  |  |
| Former vs Current | 1.11 (0.89–1.37) | 0.85 (0.67–1.08) | 0.87 (0.65–1.17) |
| **History of prior cancer** |  |  |  |
| Yes vs No | 0.92 (0.47–1.82) | 0.75 (0.36–1.55) | 1.04 (0.44–2.46) |
| **COPD** |  |  |  |
| Yes vs No | 1.25 (1.00–1.57) | 1.21 (0.95–1.53) | 1.06 (0.79–1.42) |
| **Frailty** |  |  |  |
| Frail vs Non-frail | 1.07 (0.80–1.45) | 0.88 (0.65–1.19) | 0.86 (0.59–1.25) |

Models were adjusted for age group, sex, race, ethnicity, marital status, neighborhood-level education, body mass index (BMI), smoking status, history of prior cancer, chronic obstructive pulmonary disease (COPD), and frailty, with a random intercept for site. Unknown or missing categories were included in the models but are not displayed.

Adherence was defined as completion of guideline-recommended follow-up after the index LDCT within the specified window for each Lung-RADS category: for Lung-RADS 3, completion of chest CT within 150–210 days; for Lung-RADS 4A, completion of chest CT or PET/CT within 60–120 days or biopsy within ≤45 days; and for Lung-RADS 4B/4X, completion of chest CT, PET/CT, or biopsy within ≤45 days.

**Supplementary Table S2:** Factors Influencing CT Adherence Within 11–24 Months Among Lung-RADS 1–2 and Extended Follow-Up care among Lung-RADS Positive (3, 4A, 4B/4X)

| **Variables** | **Lung-RADS Negative (1-2)** | **Lung-RADS Positive (3–4X)** |
| --- | --- | --- |
|  | **(n=21,774)*** | **(n=4,486)*** |
|  | **aOR (95% CI)** | **aOR (95% CI)** |
| **Age Group** |  |  |
| 65 and above vs Less than 65 | 1.24 (1.16–1.32) | 1.20 (1.05–1.38) |
| **Sex** |  |  |
| Female vs Male | 1.05 (0.99–1.12) | 0.93 (0.82–1.06) |
| **Race** |  |  |
| Asian vs White | 0.98 (0.88–1.10) | 0.87 (0.69–1.12) |
| Black vs White | 0.88 (0.81–0.96) | 0.90 (0.74–1.09) |
| Others vs White | 0.90 (0.68–1.19) | 0.81 (0.45–1.46) |
| **Ethnicity** |  |  |
| Hispanic vs non-Hispanic | 0.91 (0.83–1.01) | 0.94 (0.76–1.16) |
| **Marital Status** |  |  |
| Married/Living as Married vs Never Married | 1.06 (0.98–1.15) | 1.15 (0.97–1.37) |
| Previously Married vs Never Married | 1.02 (0.92–1.12) | 1.22 (0.99–1.50) |
| **Area-level Education** |  |  |
| 20–39.9% vs 0–19.9% | 1.06 (0.99–1.13) | 1.04 (0.91–1.19) |
| ≥40% vs 0–19.9% | 1.10 (0.98–1.23) | 1.10 (0.85–1.42) |
| **Body mass index (BMI)** |  |  |
| Obesity vs Normal weight | 1.03 (0.96–1.12) | 0.79 (0.67–0.92) |
| Overweight vs Normal weight | 1.10 (1.02–1.18) | 0.84 (0.72–0.98) |
| Underweight vs Normal weight | 0.91 (0.74–1.13) | 1.20 (0.86–1.69) |
| **Smoking Status** |  |  |
| Former vs Current | 1.00 (0.94–1.06) | 0.90 (0.80–1.02) |
| **History of prior cancer** |  |  |
| Yes vs No | 1.02 (0.85–1.24) | 0.79 (0.54–1.16) |
| **COPD** |  |  |
| Yes vs No | 1.25 (1.18–1.34) | 1.11 (0.98–1.26) |
| **Frailty** |  |  |
| Frail vs Non-frail | 0.84 (0.77–0.92) | 0.92 (0.78–1.09) |

Models were adjusted for age group, sex, race, ethnicity, marital status, neighborhood-level education, body mass index (BMI), smoking status, history of prior cancer, chronic obstructive pulmonary disease (COPD), and frailty, with a random intercept for site. Unknown or missing categories were included in the models but are not displayed.

For sensitivity analyses, adherence definitions were based on extended follow-up windows while maintaining the same follow-up modalities as the primary analysis. For Lung-RADS 1–2, adherence was defined as completion of any chest CT (LDCT, diagnostic CT, or PET/CT) within 11–24 months after the index LDCT. For Lung-RADS 3, adherence was defined as completion of chest CT within 120–270 days; for Lung-RADS 4A, completion of chest CT or PET/CT within 30–150 days or biopsy within ≤150 days; and for Lung-RADS 4B/4X, completion of chest CT, PET/CT, or biopsy within ≤150 days after the index LDCT.

*Analytic denominators were lower than those in the primary analysis because the sensitivity analyses used extended follow-up windows. For Lung-RADS 1–2, the sensitivity analysis was derived from the pre-final cohort shown in the study flow diagram (Figure 1; n = 22,454), after excluding participants diagnosed with lung cancer within 24 months of the index LDCT date (n = 107) or who died within 24 months (n = 573), yielding an analytic denominator of 21,774. For Lung-RADS 3–4X, the sensitivity analysis was derived from the pre-final cohort shown in Figure 1 (n = 4,558), after excluding participants who died within the defined extended follow-up period (n = 72), yielding an analytic denominator of 4,486.

**Supplementary Table 3.** Crude adherence to Lung-RADS follow-up recommendations by study site

| **Lung-RADS Group** | **Site** | **Adherent, n (%)** | **Non-adherent, n (%)** | **Total N** |
| --- | --- | --- | --- | --- |
| **Lung-RADS 1–2** | MUSC | 597 (34.45) | 1,136 (65.55) | 1733 |
|  | KPSC | 3,738 (21.98) | 13,268 (78.02) | 17006 |
|  | Florida | 439 (13.32) | 2,856 (86.68) | 3295 |
|  | Overall | 4,774 (21.67) | 17,260 (78.33) | 22034 |
| **Lung-RADS 3–4X** | MUSC | 112 (55.17) | 91 (44.83) | 203 |
|  | KPSC | 1,009 (26.14) | 2,851 (73.86) | 3860 |
|  | Florida | 167 (36.70) | 288 (63.30) | 455 |
|  | Overall | 1,288 (28.51) | 3,230 (71.49) | 4518 |

Percentages are calculated within each study site.
